# Morphometry of the thyroid cartilage, epiglottis and piriform sinus: An anatomical study

**DOI:** 10.12688/f1000research.144481.2

**Published:** 2024-04-15

**Authors:** Rajanigandha Vadgaonkar, Ashwin R. Rai, Chettiar Ganesh Kumar, B.V. Murlimanju, Mangala M. Pai, Latha V. Prabhu, Amit Agrawal

**Affiliations:** 1 Department of Anatomy, Kasturba Medical College, Mangalore, Manipal Academy of Higher Education, Manipal, Karnataka, 576104, India; 2 Department of Neurosurgery, All India Institute of Medical Sciences, Bhopal, Madhya Pradesh, 462020, India

**Keywords:** endoscopy, morphometry, otorhinolaryngology, piriform sinus

## Abstract

**Background:**

The goal was to measure the piriform sinus, epiglottis, and thyroid cartilage in our sample population.

**Methods:**

This study included 22 adult embalmed cadavers available in the Department of Anatomy. Dimensions were measured using a digital Vernier caliper.

**Results:**

The mean height of the thyroid laminae was 27 ± 1.4 mm at the right side and 25.5 ± 1.4 mm at the left side. Mean width of the thyroid lamina was 27.1 ± 1.3 mm at the right side and 27.4 ± 0.9 mm at the left side. The mean thickness of thyroid cartilage was found to be 4.4 ± 0.4 mm and 3.9 ± 0.5 mm over the right and left sides. The mean length, width and thickness of the epiglottis were 29.1 ± 0.5 mm, 22.2 ± 0.6 mm and 2.6 ± 0.3 mm correspondingly. The height, width and thickness of the right piriform sinus were 25 ± 0.5 mm, 14.2 ± 0.5 mm and 12.6 ± 0.5 mm, the same parameters were 25.3 ± 1.3 mm, 15.1 ± 0.7 mm and 13.3 ± 0.4 mm for the left side.

**Conclusions:**

The height and thickness of the thyroid cartilage were greater on the right side than on the left side (p<0.05). It was statistically observed that the width and thickness were greater on the left side than on the right side (p < 0.05). The data about the height, width and thickness of the thyroid cartilage, epiglottis and piriform sinus are essential during the laryngeal and other neck surgeries. They guide in the preoperative positioning, predicting the difficulty of intraoperative exposure and retractor pulling.

## Introduction

The larynx is a complex organ for voice production and respiration, which is supported by a series of cartilages, membranes, muscles, and joints. They bring about movement of vocal cords with a considerable range of mobility. ^1^ Apart from the phonating mechanism, it also serves as a sphincter or watch dog of the lower respiratory tract. Knowledge of laryngeal anatomy is important for various professionals, such as phoniatricians, speech therapists, oncologists, and otorhinolaryngologists. Understanding the laryngeal framework is mandatory for constructing biomechanical models for voice disorders that require accurate laryngeal dimensions. The thyroid cartilage forms a bulky structure in front of the laryngeal wall that protects the highly vibrating vocal cords located directly behind it. Because of its strength and power of resistance, it provides valuable protection to the laryngeal architecture. It has two quadrilateral laminae that are fused anteriorly in the lower two thirds leaving a ‘V’ shaped laryngeal notch superiorly. The fused anterior borders form a subcutaneous thyroid angle (laryngeal prominence) that is more prominent in males than in females. Being a hyaline cartilage, its ossification is not uncommon to the extent that, in old age, the cartilage may fracture on lateral compression or may compress backward against the vertebral column, as in strangulation or hanging. Injury to this cartilage warrants immediate attention, as subsequent laryngeal edema may lead to respiratory impairment. ^1^

The epiglottis is shaped like a leaf, projecting into the lumen of the pharynx, posteroinferior to the tongue, and hyoid bone. It is an elastic cartilage belonging to the larynx. In animals, the epiglottis serves the additional function of olfaction as well. ^2^ It develops from the 4th pharyngeal arch. ^3^^,^^4^ There is scant information in the literature about the dimensions of the epiglottis in adults. However, cadaveric studies are scarce, particularly in the Indian population. The morphometric data of the epiglottis are clinically important as there are changes in the dimensions of the epiglottis in epiglottitis.

Anatomically, the piriform sinus is a hidden area, the malignancies of which would initially cause fewer symptoms, and the patient often presents late to the hospital. It has richer lymphatic drainage, which drains into the jugulodigastric lymph nodes. Malignancy in the piriform sinus can cause distant metastasis with a higher frequency. The piriform sinus is a part of the hypopharynx; however, it is more studied along with the larynx. The piriform sinus is pear shaped space form the cavity of laryngopharynx along with the ventricle of larynx. ^5^ They are thought to add to the acoustic uniqueness of the voice. ^6^ However, they are important because of the capacity to store the food and liquid for the temporary period. ^7^ If there is any obstruction in the food passage, due to the irritation in the laryngopharynx, pooling of saliva occurs in the piriform fossa, which is known as ‘Jackson’s sign’. During food swallowing, particles such as chicken and fish bones could be impacted within the piriform sinus and may lead to dysphagia. This is an indication for endoscopic removal under general anesthesia. ^8^ The management of entrapped foreign bodies by endoscopic removal requires clinical training and skill. Inadvertent removal of the foreign body and inappropriate manipulation can cause injury to the internal laryngeal nerve, which is present deep in the piriform sinus. Injury to this nerve can cause anesthesia of the larynx above the level of the vocal cords, and may lead to the entry of food particles inside the larynx. If this continues, food particles may pass to the trachea and lungs, the accumulation of which can manifest as aspiration pneumonia. It has been reported that, the dimensions of piriform fossae have impact in the pitch of the voice particularly in the singers. ^5^ Due to all these implications, the anatomical knowledge of piriform sinus is essential to the anaesthesiologist and otorhinolaryngologist.

Data on the dimensions of the thyroid cartilage, epiglottis, and piriform fossa are essential for anesthesiologists during laryngoscopy and endotracheal intubation. However, they are scarce in scientific literature. Few studies have measured the thyroid cartilage, piriform fossa, and epiglottis in our sample population. In this context, the goal of this study was to determine the dimensions of the thyroid cartilage, epiglottis, and piriform sinus in embalmed cadavers of an Indian sample population.

## Methods

This anatomical study comprised of 22 adult embalmed human cadavers; the sample size was calculated as described in literature. ^9^ A digital Vernier caliper (Mitutoyo, Japan, 0-150 mm 500-196) was used to perform the measurements. height, width, and thickness of the thyroid cartilage, epiglottis, and piriform fossa were also measured. The measurements were taken at their maximum diameter, and for the thyroid cartilage and piriform fossa, readings were taken separately over the right and left sides. Specimens were excluded if there were any associated pathological changes. The data are presented as mean ± standard deviation, and the side-based comparison of the right and left sides was performed using the paired t-test. The most recent version of the SPSS (version 27) software was used for statistical analysis. Sex-based comparisons and age-wise segregation were not performed in the present study.

**Figure 1.**
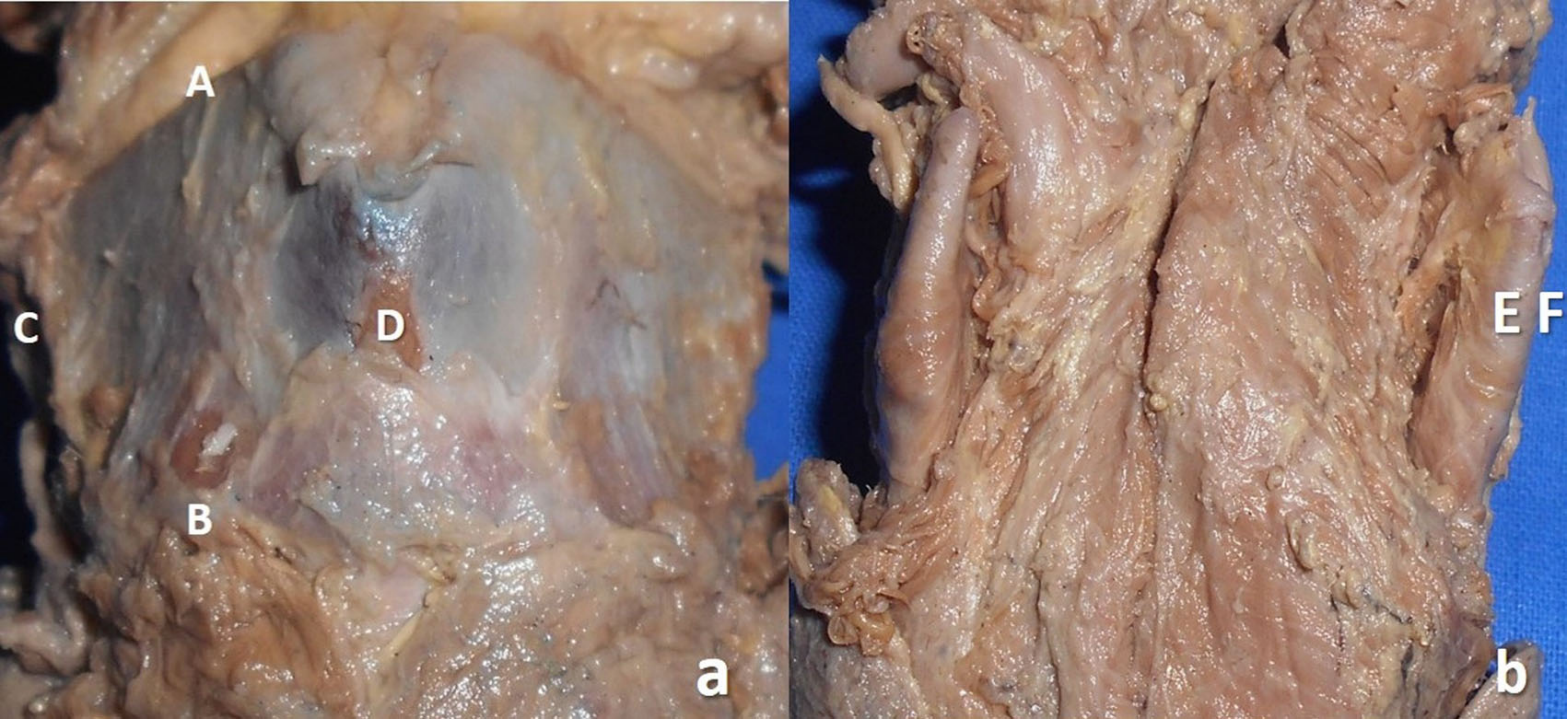
Morphometry of the thyroid cartilage of larynx in the cadaveric specimen. a. Photograph of the ventral view of thyroid cartilage showing the measurement of height (AB) and width (CD); b. dorsal aspect of the thyroid cartilage showing the measurement of thickness (EF*).

**Figure 2.**
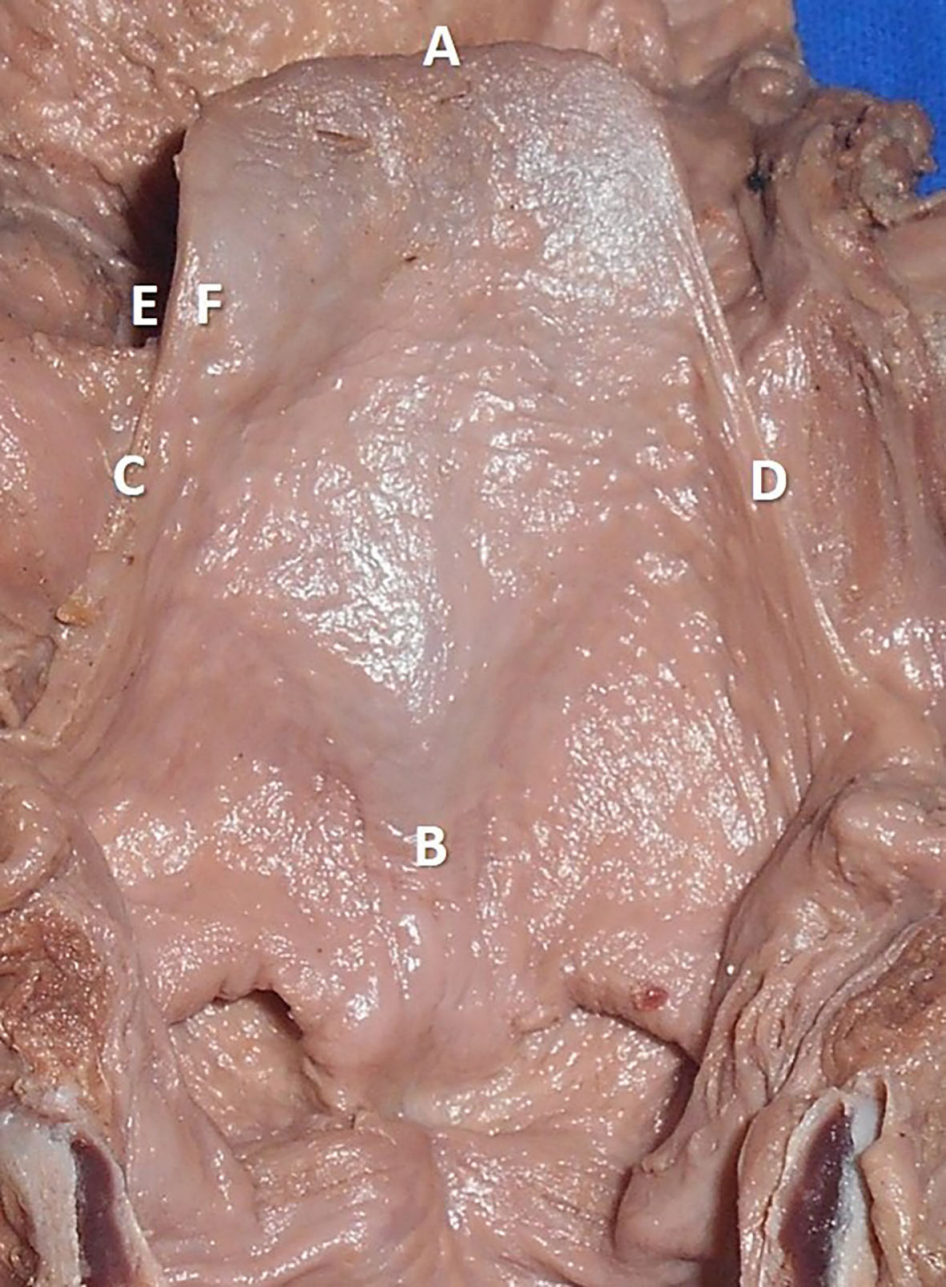
The cadaveric larynx showing the epiglottis. The measurements are represented as AB: length of the epiglottis, CD: width of the epiglottis, EF: thickness of the epiglottis.

**Figure 3.**
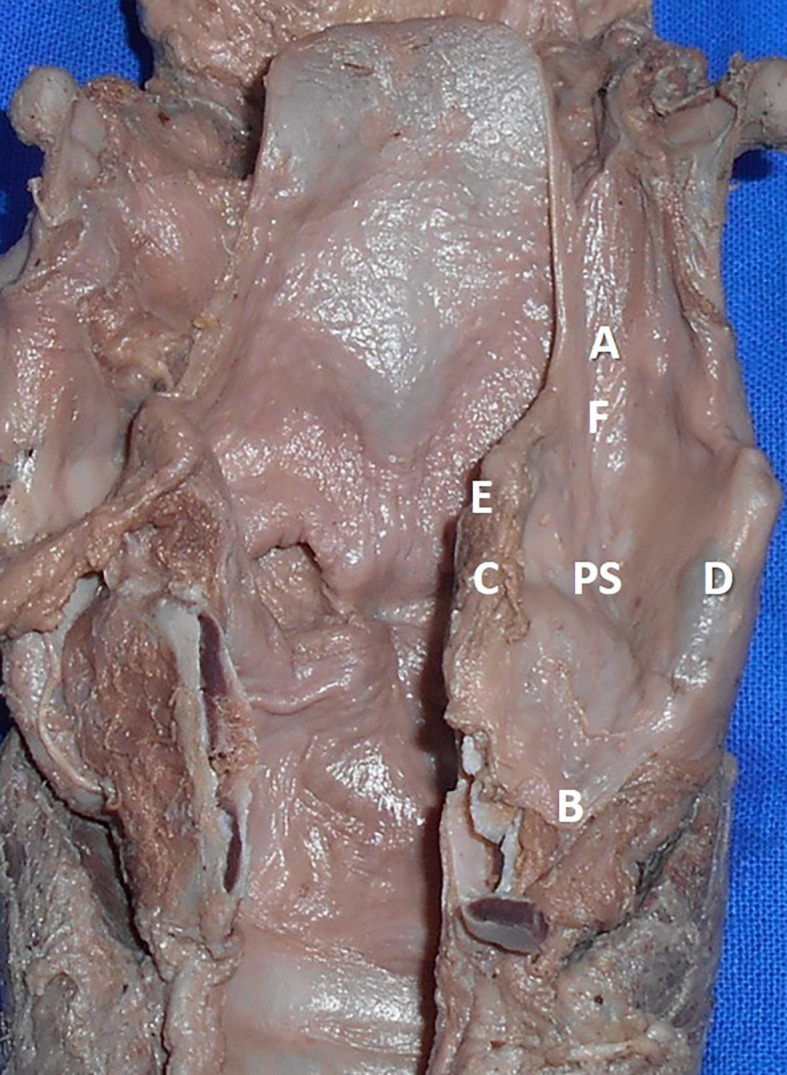
The cadaveric piriform sinus (PS). The measurements performed in this study are represented as AB: supero inferior diameter of the piriform sinus, CD: medio lateral diameter of the piriform sinus, EF: antero posterior diameter of the piriform sinus.

The measurements performed in the thyroid cartilage, epiglottis of the larynx, and piriform fossa in the present study are shown in the represented diagrams ( Figures 1, 2, and 3, respectively).

This study was approved by the institutional ethics committee of our institution (Approval Committee Name: Institutional Ethics Committee, Kasturba Medical College, Mangalore, Approval Number: IEC KMC MLR: 02/2022/61, dated 17.02.2022). We confirm that written informed consent was obtained from the body donors to conduct medical research on their bodies upon death. The protocol for this anatomical study has already been published with the dx.doi.org/10.17504/protocols.io.x54v92m8ml3e/v1.

## Results

The morphometric data of the thyroid cartilage obtained in the present study are presented separately for the right and left sides in Table 1. The mean height of the thyroid laminae was 27 ± 1.4 mm on the right side and 25.5 ± 1.4 mm on left side. The mean width of the thyroid lamina was 27.1 ± 1.3 mm on right side and 27.4 ± 0.9 mm on the left side. The mean thickness of thyroid cartilage was found to be 4.4 ± 0.4 mm and 3.9 ± 0.5 mm on right and left sides. The height and thickness were greater on the right side than on the left side. The difference was statistically significant (p<0.05). There was no statistically significant difference observed with respect to the width (p>0.05). The dimensions of the epiglottis obtained in this study are listed in Table 2. The mean length, breadth and the thickness of the epiglottis were 29.1 ± 0.5 mm, 22.2 ± 0.6 mm and 2.6 ± 0.3 mm respectively.

**Table 1.** Sidewise comparison of the morphometric data of the thyroid lamina (n=22).

Dimension	Right side (n=22)	Left side (n=22)
Height *	27 ± 1.4 mm	25.5 ± 1.4 mm
Width	27.1 ± 1.3 mm	27.4 ± 0.9 mm
Thickness *	4.4 ± 0.4 mm	3.9 ± 0.5 mm

Mean ± standard deviation; right vs. left comparison; paired t-test.

* Significance, p > 0.05.

**Table 2.** Morphometric data of the epiglottis (n=22).

Epiglottis	Mean ± standard deviation
Length	29.1 ± 0.5 mm
Width	22.2 ± 0.6 mm
Thickness	2.6 ± 0.3 mm

The dimensions of the piriform fossa obtained in the present study are given separately for the right and left sides in Table 3. The height, width and thickness of the right piriform sinus were 25 ± 0.5 mm, 14.2 ± 0.5 mm and 12.6 ± 0.5 mm respectively. The same parameters were 25.3 ± 1.3 mm, 15.1 ± 0.7 mm and 13.3 ± 0.4 mm for the left side. It was statistically observed that the width and thickness were greater on the left side than on the right side (p < 0.05). No significant difference was observed in the height of the piriform fossa between the right and left sides (p > 0.05).

**Table 3.** Dimensions of the piriform fossa (n=22).

Dimension	Right side (n=22)	Left side (n=22)
Height	25 ± 0.5 mm	25.3 ± 1.3 mm
Width ^*^	14.2 ± 0.5 mm	15.1 ± 0.7 mm
Thickness *	12.6 ± 0.5 mm	13.3 ± 0.4 mm

Mean ± standard deviation; right vs. left comparison; paired t-test.

* Significance, p > 0.05.

## Discussion

The knowledge of laryngeal anatomy has a range of applications including in clinical practice when we examine a patient to make a diagnosis, and whenever he carries out treatment in the form of a manipulation or an operation as well as for professionals (such as singers) when a speech therapist can explore the relevant anatomy for voice training. ^10^ The increasing frequency of surgical, radiological, and electrophysiological procedures performed over the larynx has led to the importance of anatomical dimensions of the larynx and its cartilages. ^9^^,^^11^ It cannot be overstated that morphometric data are essential, and there is depth knowledge that can facilitate procedures such as intubation, stenting, and endoscopic procedures of the upper aerodigestive tract. ^12^ The measurements of cartilages of the larynx are also essential to manage the subglottic and post-intubation stenosis of the lower respiratory tract. ^13^ The morphometry of the cartilages of the larynx can also be enlightening in the planning of surgical interventions of the larynx. It also has implications for placing the electrodes during the electromyography of the larynx and the interpretation of CT and MRI scans of the larynx. ^9^

Studies on laryngeal cartilage measurements have proven to be a boon in the fields of laryngeal anthropometry, physiology, imaging, and surgery. Hajiiannaou *et al*. ^14^ reported that a rotated thyroid cartilage with a dislocated superior cornu protruding in the pyriform fossa of the same side may lead to globus pharyngeus, dysphagia, and odynophagia, thereby stressing the importance of laryngeal symmetry. Eckel *et al*. ^9^ reported the average height of thyroid lamina to be 27.4 ± 2.47 mm over the right and 27.6 ± 2.32 mm over the left side in adult male cadavers. In the same study the average measurements in females were 22.2 ± 2.85 mm over the right side and 22.1 ± 2.67 mm over the left side, which was seen to be a little less. In a study conducted by Zrunek *et al*., ^15^ there was a 10% to 30% increase in the dimensions of laryngeal cartilage in males when compared to females. Park *et al*. ^16^ reported that the dimensions of the thyroid cartilage can assist in sex determination. Similar findings have also been reported by Ajmani *et al*. ^17^ and Lang *et al*. ^18^ In a study done by Jain and Dhall, ^11^ most of the dimensions of the thyroid and cricoid cartilage were higher in males than in females. They also reported obliquity of the thyroid laminae in both the sagittal and horizontal planes, thereby contributing to the increased angulation in females. They also observed asymmetry in the thyroid angle in 25% of the specimens, which deviated on either the right or left side. Kaur *et al*., ^19^ in a study conducted in 30 cadaveric adult larynges, reported similar findings of increased parameters among men. The present study compared the data of the right and left thyroid cartilages. However, sex-based comparisons were not performed in our study.

The dimensions of the epiglottis are essential for anesthesiologists and ear nose throat surgeons. The length and width of epiglottis in an infant has been reported to be 13.15 ± 0.44 mms and 8.92 ± 0.3 mms respectively. ^20^ The ultrasound thickness of epiglottis measured 2.4 ± 0.2 mm in a Chinese population. ^21^ According to American sonographic research by Werner *et al*., ^22^ the epiglottic thickness was 2.39 ± 0.15 mm. The present study from the cadaver specimen reported a mean thickness of 2.6 ± 0.3 mm, which is similar to these reports. Werner *et al*. ^22^ endorsed that the epiglottis is thicker in the male than in other structures of the body. The present study could not perform a gender-based comparison, which is a limitation of this study.

Morphometric dimensions of the piriform fossa are educative to doctors of anesthesiology and laryngeal surgery because this is the location of entrapment of foreign particles such as fish and chicken bones during eating. The foreign body must be removed endoscopically, as this may cause infection and difficulty in swallowing. The morphology of the piriform fossa is also important to determine its acoustic properties. ^23^ In this perspective, dimensions of the piriform sinus will help in the manufacture of the flexible fiber optic endoscopic instruments, which can match to that particular population. The present study provides morphometric data for the piriform sinus on the right and left sides. The length, width, and thickness are also provided. However, the data of the present study cannot be considered as a morphological database of our sample population, as the sample size was small. However, this sample size cannot represent the population subgroup of the entire country.

## Conclusions

The present anatomical research presents the morphometric data of the thyroid cartilage, epiglottis, and piriform fossa in cadaveric samples of our sample population, which will help compare them with other populations. These data will guide the manufacture of endoscopic devices and endotracheal tubes with respect to our sample population. The height and thickness of the thyroid cartilage were greater on the right side than on the left side (p<0.05). It was statistically observed that the width and thickness were greater on the left side than on the right side (p<0.05). The data about the height, width and thickness of the thyroid cartilage, epiglottis and piriform sinus are essential during the laryngeal and other neck surgeries. They guide in the preoperative positioning, predicting the difficulty of intraoperative exposure and retractor pulling. However, the limitation of this study is the small sample size; the data will only be accurate with a larger sample size.

## Data Availability

Figshare: Underlying data for ‘Morphometry of the thyroid cartilage, epiglottis and piriform sinus, an anatomical study’, https://doi.org/10.6084/m9.figshare.24455329.v1 Data are available under the terms of the Creative Commons Zero “No rights reserved” data waiver (CC0 1.0 Public domain dedication).
