# Supplementary material for: Morphometry of the thyroid cartilage, epiglottis and piriform sinus: An anatomical study
Source: F1000Res. 2024 Apr 15;13:115. Originally published 2024 Feb 19. [Version 2] doi: 10.12688/f1000research.144481.2 (PMC11249526; doi:10.12688/f1000research.144481.2)
Supplement: Peer review history

# Reviewer report: Reviewer response for version 2

**DOI:** 10.5256/f1000research.164652.r292190

**Published:** 2024-07-15

**Authors:** Ilhem Charfeddine, Mohamed Amin Chaabouni

**Affiliations:** 1 University of Sfax, Sfax, Tunisia; 2 Department of otorhinolaryngology-Head and Neck Surgery, Habib Bourguiba Hospital (Ringgold ID: 518993), Sfax, Sfax, Tunisia

Keywords:

 Add: epiglottis, thyroid cartilage

 Delete: endoscopy

 Introduction:

 Too much unnecessary detail, you need to be clear about the problematic that led to the study.

 Methods/Results:

 Specify whether measurements were made on male or female cadavers.

 Specify how epiglottis width was calculated: 

 1. In Figure 2, the width was measured from the right aryepiglottic fold to the left, which does not accurately represent the width of the cartilage itself.

 2. Specify that this is the greatest width.

 3 As shown in the figure, measurement after posterior opening of the cricoid cartilage may overestimate the exact width. This has different clinical implications

Is the work clearly and accurately presented and does it cite the current literature?

Yes

If applicable, is the statistical analysis and its interpretation appropriate?

Yes

Are all the source data underlying the results available to ensure full reproducibility?

Yes

Is the study design appropriate and is the work technically sound?

Partly

Are the conclusions drawn adequately supported by the results?

Yes

Are sufficient details of methods and analysis provided to allow replication by others?

Yes

Reviewer Expertise:

Head and neck surgery

We confirm that we have read this submission and believe that we have an appropriate level of expertise to confirm that it is of an acceptable scientific standard, however we have significant reservations, as outlined above.

# Reviewer report: Reviewer response for version 2

**DOI:** 10.5256/f1000research.164652.r301063

**Published:** 2024-07-15

**Authors:** Pradip Chauhan

**Affiliations:** 1 Department of Anatomy, All India Institute of Medical Sciences, Rajkot, Gujarat, India

Authors have tried to cover all important aspect regarding the title.

 The results and discussion would be more valuable with the following added points.

1. Result and analysis on the base of Gender of the cadaver. 
2. Consideration of the stature of the cadavers to minimize the compounding factors like height.
3. Increasing the sample size to 30
4. Including the maximum diameter of the laryngeal inlet as a part of parameter

Is the work clearly and accurately presented and does it cite the current literature?

Yes

If applicable, is the statistical analysis and its interpretation appropriate?

Yes

Are all the source data underlying the results available to ensure full reproducibility?

Yes

Is the study design appropriate and is the work technically sound?

Yes

Are the conclusions drawn adequately supported by the results?

Yes

Are sufficient details of methods and analysis provided to allow replication by others?

Yes

Reviewer Expertise:

Virtual reality, embryology, Gross human anatomy, medical education. digital health, artificial intelligence, Microgravity, histology, cardiovascular anatomy, neuroanatomy

I confirm that I have read this submission and believe that I have an appropriate level of expertise to confirm that it is of an acceptable scientific standard.

# Reviewer report: Reviewer response for version 1

**DOI:** 10.5256/f1000research.158285.r252283

**Published:** 2024-03-28

**Authors:** José Aderval Aragão, Deise Maria Furtado de Mendonça

**Affiliations:** 1 Morphology, Federal University of Sergipe (Ringgold ID: 74391), São Cristóvão, State of Sergipe, Brazil; 2 Universidade Federal de Sergipe, São Cristóvão, State of Sergipe, Brazil

We conducted a thorough review of the manuscript titled "Morphometry of the thyroid cartilage, epiglottis, and piriform sinus: An anatomical study." The study aimed to determine the dimensions of the thyroid cartilage, epiglottis, and piriform sinus in embalmed cadavers from an Indian sample population. The paper offers a detailed morphological and morphometric analysis and establishes significant clinical correlations.

 The findings are valuable for professionals in various fields, including otolaryngologists, oncologists, intensive care physicians, anesthesiologists, speech therapists, and phoniatricians, as well as for comparisons with studies in other populations. The results are succinct, and the discussion section effectively places the findings in the context of existing literature.

 Unfortunately, a comparative analysis between males and females was not feasible, as acknowledged by the authors themselves as a study limitation. Nevertheless, the absence of this comparison does not diminish the significance of the presented data.

 Some minor points for improvement include:

 - Correcting the word "width" in the description of Figure 2.

 - The conclusions should address the differences between the right and left sides in terms of length, width, and thickness, and clinically discuss the implications of these measurements.

Is the work clearly and accurately presented and does it cite the current literature?

Yes

If applicable, is the statistical analysis and its interpretation appropriate?

Yes

Are all the source data underlying the results available to ensure full reproducibility?

Yes

Is the study design appropriate and is the work technically sound?

Yes

Are the conclusions drawn adequately supported by the results?

Yes

Are sufficient details of methods and analysis provided to allow replication by others?

Yes

Reviewer Expertise:

Clinical Anatomy and Medicine

We confirm that we have read this submission and believe that we have an appropriate level of expertise to confirm that it is of an acceptable scientific standard.

# Response

**Published:** 2024-04-04

**Authors:** B.V. Murlimanju

**Affiliations:** ANATOMY, Manipal Academy of Higher Education, Mangalore, Karnataka, India

We thank the reviewer for the expert opinion.

 As per the suggestion, the conclusions were revised. The typographical error was corrected.
